# Nanoscale Depth Profiling of Optoelectronic Devices Using Deep-UV LIBS

**DOI:** 10.1021/acsomega.5c08550

**Published:** 2025-11-21

**Authors:** Atchutananda Surampudi, Mool. C. Gupta

**Affiliations:** Charles L. Brown Department of Electrical & Computing Engineering, 744402 University of Virginia, Charlottesville, Virginia 22904, United States

## Abstract

Accurate elemental nanoscale depth profiling is vital for semiconductor junctions, optical coatings, and thin-film photonic devices. While Secondary Ion Mass Spectrometry (SIMS) is capable of providing nanoscale elemental depth profiling, it requires bulky and complex instrumentation and is not portable for real-time process monitoring. Alternatively, the technique of Laser-Induced Breakdown Spectroscopy (LIBS) has been shown to provide sensitive detection of elements; however, its depth resolution is limited to approximately μm, hindering nanoscale profiling. Here, we demonstrate nanoscale elemental depth profiling (∼10s of nm) using deep UV LIBS under ambient conditions. Using a fiber-coupled 266 nm wavelength (UV–C) pulsed laser, the system achieves an ablation depth as low as ∼20–25 nm per pulse, enabling high-resolution profiling, while maintaining an elemental detection sensitivity in the parts-per-million range. Depth profiling of a silicon photovoltaic device’s diffused silicon PN junction reveals a clear boron dopant profile signal within ∼650 nm, aligning with expected emitter diffusion depths. Similarly, the depth profiling of a dielectric mirror reveals the nanoscale alternating layers of optical coatings of Ta_2_O_5_ and SiO_2_ elemental profiles, each measuring respectively ∼100 and ∼145 nm. Furthermore, with deep UV single pulse ablation, the detection sensitivity of a nanoscale thin (∼1–2 nm) native oxide film on top of a silicon wafer is also demonstrated. The developed instrument for LIBS is an optical head packaged in a 3 × 2 × 1.5 cm^3^ (24 g) compact head, allowing autofocusing for best ablation, while employing a custom ball lens for tight spot focusing and efficient collection of the plasma emission light. This is an attractive characterization approach for real-time nanoscale elemental mapping and depth profiling of optical/electronic devices, eliminating the need for vacuum or extensive sample preparation, while allowing for the demonstration of a portable device that can operate under ambient conditions.

## Graphical abstract

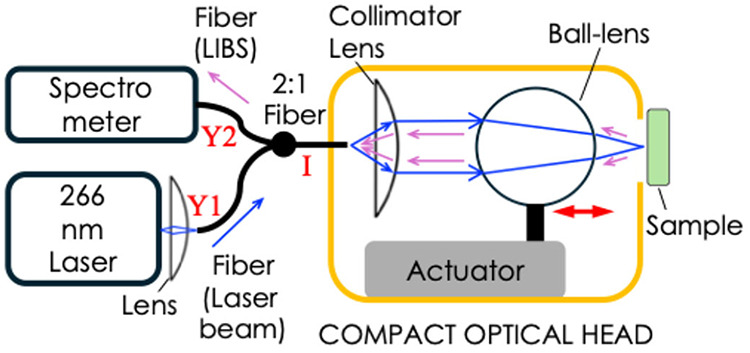

## I Introduction

Accurate, nanoscale elemental depth analysis is critical to the design and fabrication of multilayer semiconductor junctions, optical coatings, and thin-film optoelectronic devices. Multilayer thin films are used in sensors, optical antireflection coatings, optical filters, LEDs, photonic crystals, displays, integrated photonic chips, and semiconductors etc. Multilayer thin films allow the manipulation of light and other properties at the nanoscale. Secondary Ion Mass Spectrometry (SIMS) has long been the standard for nanoscale elemental profiling, routinely achieving ∼10s of nm depth resolution and subppm detection limits. However, it requires ultrahigh vacuum, costly instrumentation, and is constrained by immobile benchtop operation.^1,2^ These constraints preclude real-time, in-line monitoring during device fabrication or field deployment, motivating the search for portable alternatives that could operate under ambient conditions.

Laser-Induced Breakdown Spectroscopy (LIBS) is based on the detection of the photons emitted by the atomized sample in a plasma generated during laser ablation. LIBS offers rapid, multielement analysis under atmospheric conditions with minimal sample preparation, making it attractive for depth profiling of layered materials.^3^ Conventional LIBS implementations typically use visible or near-infrared excitation, producing ablation craters with depths in the order of 0.5–1 μm per pulse.^4^ Since LIBS causes only ∼micron-scale lateral damage, it has been deployed in harsh environments, such as in fusion reactors, to perform multielement multilayer depth profiling.^3,4^ LIBS has been successfully applied to depth profiling of photovoltaic absorbers (e.g., Cu­(In,Ga)­Se_2_ films),^5,6^ doped semiconductor layers,^7^ and multilayer optical coatings,^8^ demonstrating its versatility across metals, semiconductors, and dielectrics. LIBS has also repeatedly shown to detect trace elements at low parts-per-million levels and can detect dopant concentrations relevant to semiconductor processing.^9,10^ Experimental methods to lower the detection limits of LIBS have included spectral selection of molecular bands, temporally gated detection, and sample pretreatments, all of which have been shown to reduce matrix interference and improve the precision for trace analysis.^11^ Studies specifically targeting boron or other light dopants report practical demonstrations on metals, alloys and solids that are directly relevant to dopant profiling. Selective enhancement of the B I line produced subppm limits of detection with LIBS in steels and superalloys.^12^ Recent quantitative LIBS work with optimized sample handling has achieved ppb–ppm level performance for low-Z elements in solids.^13^ These demonstrations establish a realistic technical basis for applying LIBS to trace elemental detection, particularly in semiconductors.

Depth-resolved LIBS with sequential single-spot ablation has been applied across a broad set of layered and graded materials, including metal coatings, ceramic and oxide films, multilayer polymer systems, cultural-heritage paint stratigraphy, and engineered multilayer systems.^3^ Early industrial demonstrations showed online monitoring of galvanized steel and coated sheet processes with layer-by-layer LIBS measurements, and subsequent work also resolved metal diffusion. Thin metal films on silicon, alumina coatings, and tungsten coatings on structural alloys, all use LIBS depth sequences to obtain layer composition information and approximate thickness (∼μm).^14–17^ Polymer multilayers and other organic thin films have also been depth-probed by LIBS to map composition through layered stacks.^18–20^ Taken together, these studies justify LIBS as a well-established functionality for depth profiling of multilayered materials.

Despite these advances, the current LIBS techniques, particularly under ambient conditions, are limited in resolution to ∼μm. As a result, the elemental/dopant profiles of diffused semiconductor junctions which are only limited to a few 100s on nm, cannot be resolved at the nanoscale,^3^ therefore limiting their alternative use with SIMS. While there have been efforts in performing nanometric profiling, such as—using low pressure environments, use of a flat energy profiles of laser beams; these efforts suffer through a complexity of hardware which limits the use of LIBS for in situ depth profiling applications, particularly in semiconductor fabrication laboratories.^21,22^

LIBS with shorter wavelength excitation in the UV is shown to reduce the optical penetration depth due to stronger absorption for most materials and improve the interface delineation in metallic multilayers.^22^ Similarly, for elemental mapping of battery electrodes, UV LIBS has been demonstrated to achieve a resolution of ∼200 nm, suggesting that optimization of wavelength could improve the depth resolution.^23–25^ Furthermore, optimizing the fluence of a UV nanosecond laser preventing the effect of superheating has been shown to achieve nanoscale ablation ∼10–80 nm per pulse.^26,27^ Beyond wavelength selection, miniaturized and fiber-delivered LIBS have enabled in situ, real-time elemental mapping in remote surfaces,^28^ plasma reactors,^29^ and recently, for mixture detection in planetary exploration.^30^ These efforts show the importance of integrating LIBS into hand-held platforms coupled with UV wavelength-based probing.

Realizing that most materials have optical absorption depths in the order of a few 10s of nanometers with shorter wavelengths in the UV, it has motivated us to further explore the benefits of LIBS over the deep-UV range with the aim of achieving nanoscale depth resolution. For example, the absorption depth in silicon for a 266 nm laser wavelength, which is in the UV–C range, is less than 10 nm. In this paper, we take a significant step further in performing LIBS with much shorter wavelengths (deep-UV 266 nm), particularly, to achieve a depth resolution in the nanoscale ∼10s of nm. Using a deep-UV (UV–C) wavelength allows us to demonstrate an ablation depth of as little as ∼20 nm per pulse while delivering rapid, layer-by-layer elemental profiles. The analysis of three specific examples of optoelectronic devices are considered: (a) the detection of the dopant profile of a diffused Si PN junction of interest in semiconductors/photovoltaics; (b) the detection of the multilayer oxide coatings of a highly reflective dielectric mirror, which is of interest in optical applications, (c) the detection of the top surface nanometer thin oxide layer on top of a silicon wafer. For the diffused junction, nanoscale mapping allows the detection of boron dopant signal decay within ∼650 nm, closely matching diffusion models, and validating the expected diffusion lengths. For the dielectric mirror, alternating layers of optical coatings of Ta_2_O_5_ and SiO_2_ are revealed, each measuring a thickness of ∼100 and 140 nm. Furthermore, for the silicon wafer, the technique is demonstrated to detect the presence of a nanoscale thin (1–2 nm) top surface oxide layer, upon spatial summing of the measured spectra. These LIBS demonstrations are performed in ambient conditions in air, using a fiber-coupled 266 nm wavelength- deep-UV compact laser coupled to an optical head packaged in a 3 × 2 × 1.5 cm^3^ in size. The compact head also incorporates a miniature ball-lens, ensuring tight spatial focusing with a 1/e^2^ beam diameter of ∼38 μm, allowing efficient UV ablation in a much smaller footprint, while also allowing efficient signal collection of the plasma emitted light. By uniting the nanoscale ablation with deep-UV and the fiber-coupled autofocus-based compact design of the optical head, we overcome the portability, resolution, and operational constraints of all prior implementations—enabling real-time, in situ dopant/elemental mapping of optoelectronic devices, particularly under ambient conditions, and therefore advancing the utility of LIBS in conducting highly resolved nanoscale depth profiling. Our approach removes vacuum needs and cuts analysis time from hours to seconds. Section 2 describes the optical layout and packaging of the compact optical head used for the experiments. Section 3 presents depth-profiling measurement results. Section 4 presents the discussions, and Section 5 presents the conclusions.

## II Experimental Setup for DUV LIBS

The schematic diagram of the experimental setup of the deep UV LIBS is shown in Figure 1. The yellow box depicts the arrangement of the components inside the packaged optical head. The laser (Crylink, 266 nm, 1.5 ns pulse width, 13 μJ pulse energy) and the spectrometer (Avantes Avaspec 4096 CL 200–310 nm) are external to the optical head, which is nonetheless compact and lightweight. The output beam of the laser is focused using a convex lens (Thorlabs LA4936, *F* = 30.1 mm), into the Y1 input-end of a Y-2:1 optical fiber (Ocean Optics, 200 μm core diameter, NA = 0.22). The light (blue color arrow) is output from the I-end of the fiber, which is fixed inside the optical head. The light inside the head is collimated using a collimating lens (Edmund Optics 88-173, 4 mm diameter, NA = 0.22), then it is focused onto the sample using a ball lens (Edmund Optics 67-388, 8 mm diameter UV fused silica). For a 4 mm size collimated beam falling on the 8 mm diameter ball lens, the light is focused with an NA = 0.31, allowing a tight focusing ∼38 μm spot size on the sample. The use of a ball lens introduces novelty in the system as it acts as a light concentrator, allowing a tight focus. The ball lens is mounted on an actuator (Newscale M3L, range 6000 μm, 1 μm minimum step size) to allow for autofocusing and position the focus on the sample. Upon laser ablation, the light from the plasma (pink color arrow) is collected back with the ball lens, which collimates the light, and which is focused back into the fiber via the collimator lens. The light is then coupled into the spectrometer via the other end of the fiber. As a note, a LIBS setup could also be realized as a free-space setup without the fiber, which, however, makes the optical components in the instrument prone to vibrations and misalignment. The 3D-printed optical head is described next, as shown in Figure 2.

**Figure 1:**
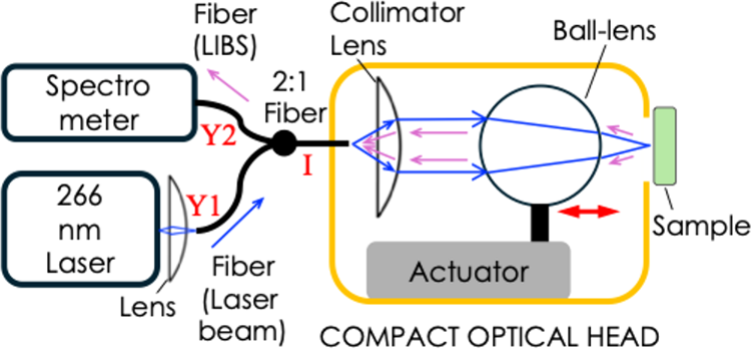
Schematic diagram of the setup.

**Figure 2:**
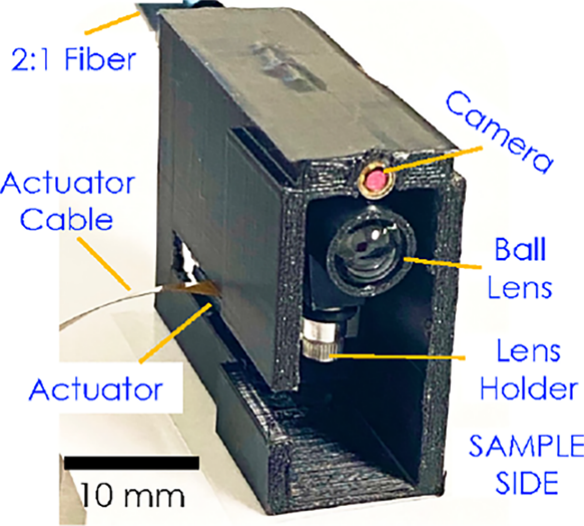
3D printed optical head.

The 3D printed unit for the optical head is shown in Figure 2. The CAD design for the optical head is included in the supplementary files as Figure S1a–d. The 3D print of the head consists of an actuator slot (to accommodate the M3L actuator), a fiber slot to accommodate the I-end of the fiber coupled with the collimator, a slot to accommodate a fiber-based visible imaging camera (Endosnake), which allows obtaining visible surface images of the sample.

The distance between the home position of the ball lens on the actuator stage to the sample side is 6000 μm, allowing a full range scan for focus. To perform LIBS, the sample would be placed close to the sample side of the optical head, and the fiber from the back of the head is used to deliver/collect the emitted light. This one-body integrated optical head packages the UV fused miniature ball lens, the actuator, the UV collimator, and the fibers into a small size of 3 × 2 × 1.5 cm^3^, with a weight of ∼24 g, therefore demonstrating compactness and hand-held operation useful for real-time monitoring. Externally, the deep-UV laser occupies a compact volume of 120 × 45 × 30 mm^3^ with a weight of 215 g, an additional power supply unit: 168 × 88 × 140 mm^3^, weighing 200 g; and the spectrometer occupies a compact volume of 95 × 65 × 20 mm^3^ with a weight of 90 g. So, overall, all three components combined would weigh ∼520 g. The laser and the spectrometer can be carried as a portable compact unit (just weighing 500 g), and the optical head can be hand-held, allowing portable detection.

## III Nanoscale Depth Profiling Measurement Results

Losses in the light coupling at the Y1-end of the optical fiber, the transmission characteristics of the optical fiber for 266 nm wavelength, and the optical reflection losses at the collimator lens and ball lens means a beam power of ∼7 mW (at 1 kHz repetition rate) is measured at the sample-side. The energy per pulse delivered at the sample-side is calculated as 7 μJ.

### a PN Junction Dopant Profiling

A commercially available diffused PN junction silicon solar cell wafer (Figure 3a), which is of interest for photovoltaic applications, is considered for demonstration. The schematic cross-section of the wafer is shown (Figure 3b). It consists of an N-type substrate (thickness ∼300 μm), on top of which P-type dopant is diffused (few 100s of nm, typically ∼650 nm), with a top insulating layer of Si_3_N_4_ (typically ∼50–80 nm).

**Figure 3:**
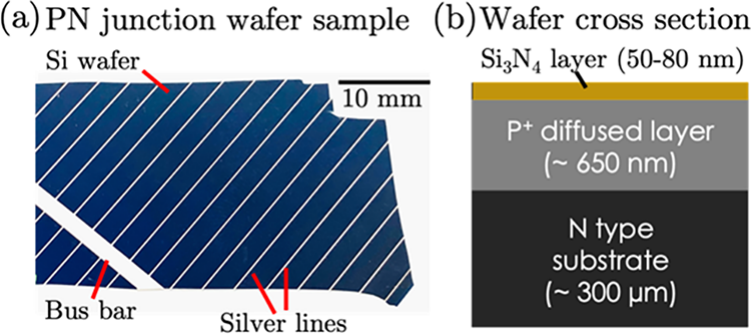
(a) Semiconductor wafer sample image obtained with the camera packaged in the optical head, (b) cross-section.

Based on measurements with reflected light, the ball lens is translated to achieve maximum signal with best focus as shown in Figure S1. At focus, the laser beam ablates the sample to make an ablation mark that is ∼25 μm wide. The ball lens is translated on the actuator to reach the focus position. To accurately determine the 1/*e* ^2^ diameter of the beam, knife-edge measurements are made of the beam at focus, as shown in Figure 4. A 1/*e* ^2^ beam diameter of ∼38 μm is measured. This means, at focus, the fluence per pulse is calculated as ∼0.617 J/cm^2^. The depth profiling experiment is performed at this focus position.

**Figure 4:**
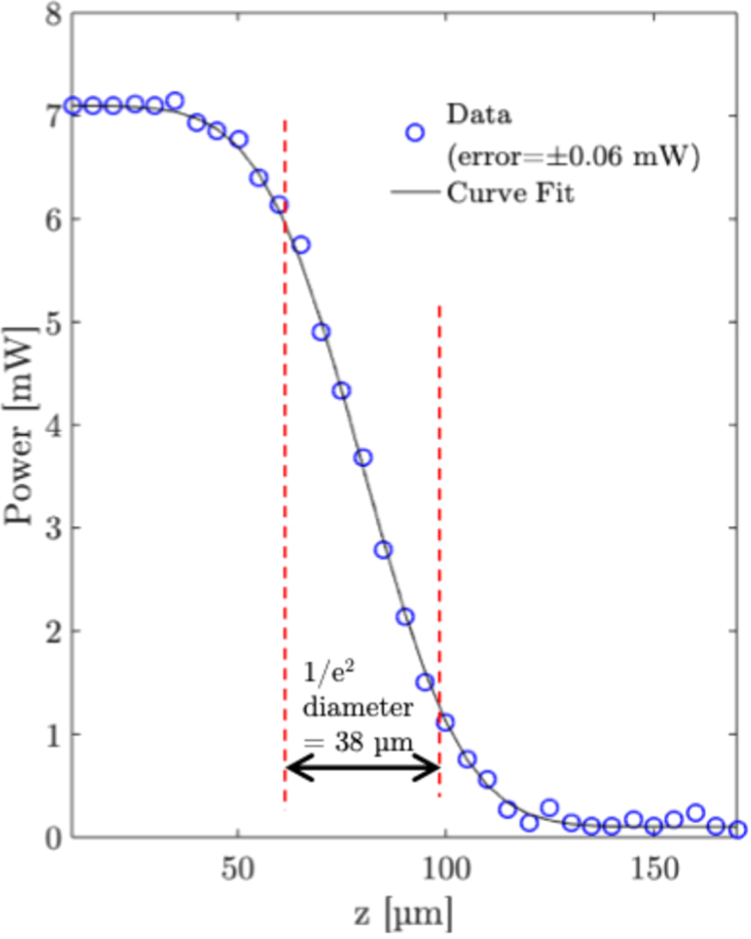
Knife edge measurements.

The laser repetition rate is set to 1 Hz, and the integration time of the spectrometer is set to 1 s (with a delay of 1 μs at the start of each scan to circumvent the laser light and capture the plasma light). A fresh sample area is considered. As the pulses sequentially impact the wafer, starting from the top layer, they create an ablation crater that becomes deeper with every laser shot. While the LIBS spectrum is recorded at each ablation shot, giving the elemental map at the given depth of ablation, the LIBS intensity gives the concentration of the element at that depth.

The recorded LIBS spectra over different numbers of pulses are shown in Figure 5. The spectra are noise filtered to detect the peaks. With 3 pulses, a boron peak at 208.9 nm wavelength was observed. As the number of pulses increases sequentially, the boron LIBS peak gradually reduces. By the 36th pulse, the LIBS peak of boron disappears. However, the phosphorus LIBS peaks at 213.6 and 214.9 nm wavelengths are measured with a further increase in the number of laser pulses. After 104 pulses, the depth of the crater formed by successive ablation is measured using an atomic force microscope (AFM, Bruker Innova) as 2.11 μm (Figure 6a). From Figure 6a, it is observed that for 12, 28, and 56 pulses, a depth of respectively 252, 580, and 1134 nm is obtained. The scanning electron microscope (SEM) surface images (Phenom, 2650X, BSD, 10 kV) are shown in Figure 6b.

**Figure 5:**
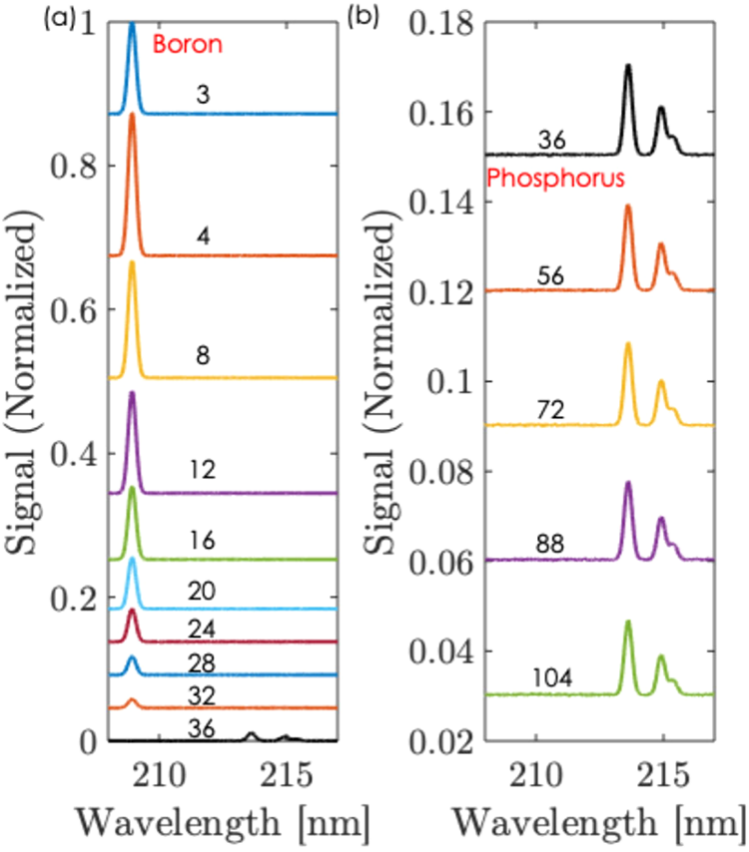
LIBS Spectrum: (a) after 3–36 pulses, (b) after 36–104 pulses.

**Figure 6:**
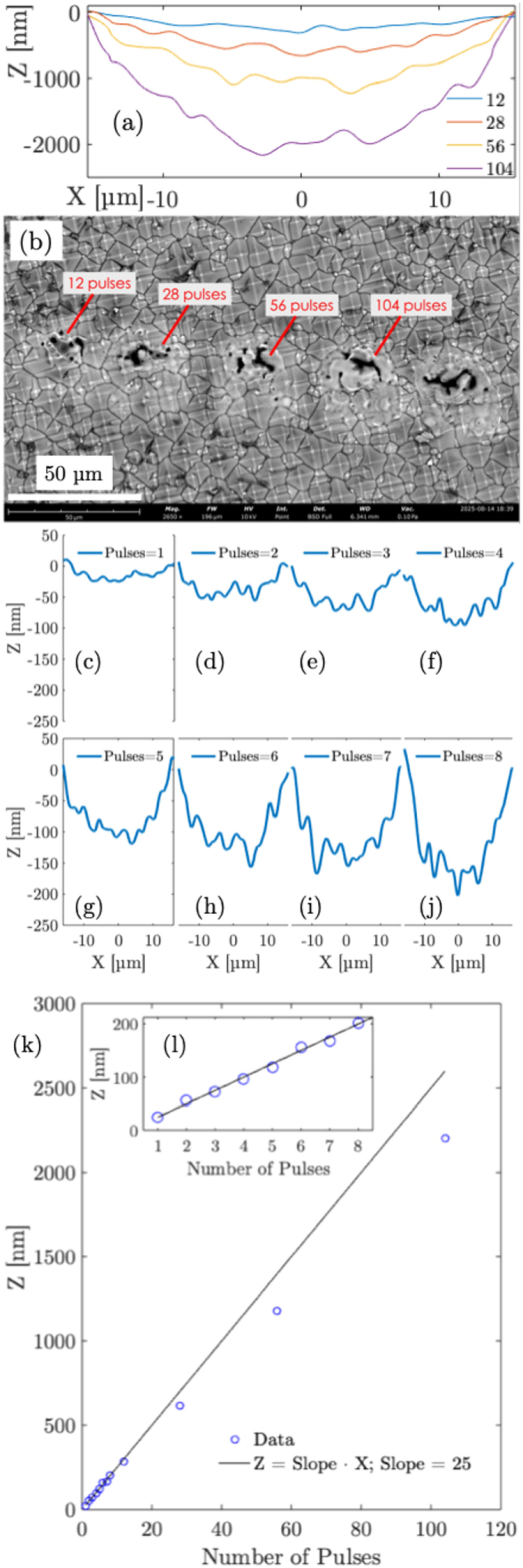
(a) Depth profiling, (b) SEM image, (c–j) Depth profiling measurements for pulses 1–8. (k–l) depth vs number of pulses.

These measurements, on an average, calculate the depth per pulse of ablation ∼20 nm. To verify this ablation depth, more measurements were made with fewer ablation shots (1–8) using the AFM. These measurements are shown in Figure 6c–j. With one pulse, a depth of ∼25.3 nm is observed. With successive pulses, 2–8, a depth of respectively 50, 74.2, 98, 121.3, 144.1, 166.3, 189.9 nm is observed. A graph of the ablation depth versus the pulse number is plotted in Figure 6k. The measurements for pulses 1–8 curve fit with a linear plot with a slope of 25 nm, as also observed in the inset in Figure 6l. For larger number of pulses, the measurements start deviating gradually showing nonlinear behavior. A nonlinear deviation is expected due to factors such as plasma shielding and beam scattering with the evolution of the crater. This means starting from ∼25 nm with fewer pulses, the depth per pulse of ablation varies between 20–25 nm in the performed experiments.

Such nanoscale shallow ablation depths with similar fluences ∼0.4–0.8 J/cm^2^ have been reported previously for boron emitter silicon solar cell PN junctions using deep-UV nanosecond laser.^26^ The reason for a shallow depth of ablation with a deep-UV nanosecond laser has been attributed previously to the use of low fluence values which do not cause superheating of the subsurface of the material.^27^

The LIBS profiling measurements are presented next. The peak intensities from the LIBS spectra are recorded for both boron and phosphorus over multiple measurements (to obtain measurement statistics and ensure repeatability), as shown in Figure S2. The average values of these measurements in Figure S2 are plotted versus the pulse number as shown in Figure 7a. The error bars represent the standard error from the mean. From Figure 7a, the boron intensity is initially detected from the third pulse (∼75–80 nm). This is due to the absence of boron in the top insulating layer of Si_3_N_4_. Beyond this insultating layer, the diffused layer starts, with the maximum intensity of boron detected at the fourth pulse (∼100 nm). Thereafter, the boron intensity reduces, which indicates the doping profile of boron in the P-type diffused layer. The boron intensity diminishes after 32 pulses, and based on the plot in Figure 6k, this depth is estimated to be ∼650 nm, which is expected for a solar-cell PN junction.^31^ It is worth noting that the observed standard error in the measurements in Figure 7a,b is ±20 counts over four repeated measurements. This arises from the spectrometer noise and shot-to-shot fluctuations in laser energy and variation in ablation volume, with smaller contributions from sample inhomogeneity. These variations in the intensity are within the experimental error and ensure the reliability of the technique.

**Figure 7:**
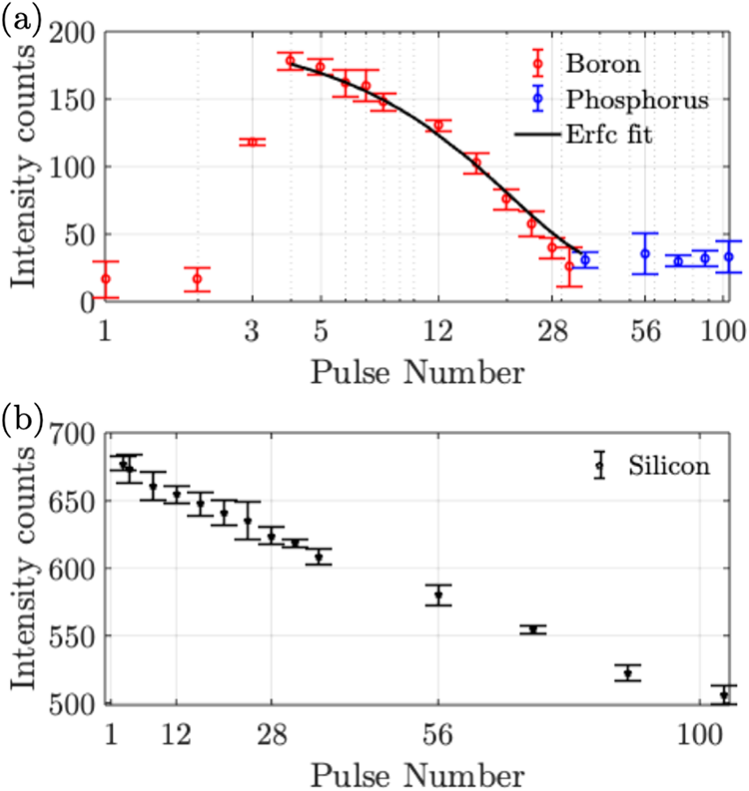
LIBS intensity vs pulse number: (a) boron and phosphorus, (b) for silicon.

To validate the results shown in Figure 7a, the boron data is curve fitted with an *erfc* function following Fick’s second law of dopant diffusion:

1 $$y_{\mathrm{fit}}=\mathrm{erfc}\left(\frac{d}{2\sqrt{D\times t}}\right)$$

where *d* is the depth of diffusion, *t* is the time of diffusion, and *D* is the temperature-dependent diffusivity, which depends on the temperature *T* as *D*_o_exp­(−*E*_a_/*kT*), where *D*_o_ is the pre-exponential factor, *E*_a_ is the activation energy for diffusion, and *k* is the Boltzmann constant. The curve fit parameters provided a temperature of 900 °C and a time of diffusion of ∼1.5 h, which are usual for solar cell manufacturing. The validity of the erfc curve fit can be observed with the error bars, which deviate within ±20 counts and which lies within the experimental error. In the case of phosphorus, the recorded intensity of phosphorus remains approximately constant, which indicates a constant doping profile of phosphorus in the N-type substrate. The absence of a phosphorus peak before the 36th pulse could be attributed to the matrix interference in the diffused layer, where boron was dominant, along with the dependence of the LIBS intensity on other parameters such as the cross section of the emission.^32^ For reference, the reduction of the intensity of the constituent silicon peak is also plotted in Figure 7b, where the peak intensity only reduces by ∼20%, which could be attributed to the scattering of the laser beam due to a different depth of ablation. While this contributes to a reduction in the recorded intensity, this is insignificant compared to the >90% reduction in intensity in boron due to the doping profile.

### b Multi-Layer Dielectric Mirror

To further demonstrate the versatility of the nanoscale depth profiling, another example from photonics, a dielectric mirror that is composed of multilayer optical coatings, was selected. In contrast to a PN junction where the diffusion profile of a trace dopant determines device performance, a dielectric mirror is a stack of thin film oxide layers which alternate every ∼100–150 nm, and where the thickness of each layer determines the mirror’s optical characteristics.

A commercially available broadband dielectric mirror was used, which shows >75% reflectivity in the 650–1000 nm NIR wavelength range, with a center wavelength of 850 nm. Such NIR mirrors have been investigated previously to have alternating layers of optical coatings, usually the oxides of tantalum and silicon.^33–35^ The alternating coatings are deposited so that the thickness Δ of each layer, with refractive index *n*, determines an optical path length *n*Δ, which equals a quarter of the central wavelength λ of the desired reflectance. For the mirror, with a central wavelength λ = 850 nm, the thicknesses of Ta_2_O_5_ and SiO_2_ layers can be calculated as, respectively, 100.95 nm (*n* = 2.105) and 145.84 nm (*n* = 1.457), which are close to the thicknesses reported for such mirrors previously.^34^ The KLA software^36^ was used to simulate the reflectance spectrum of such a mirror, shown in Figure 8a.

**Figure 8:**
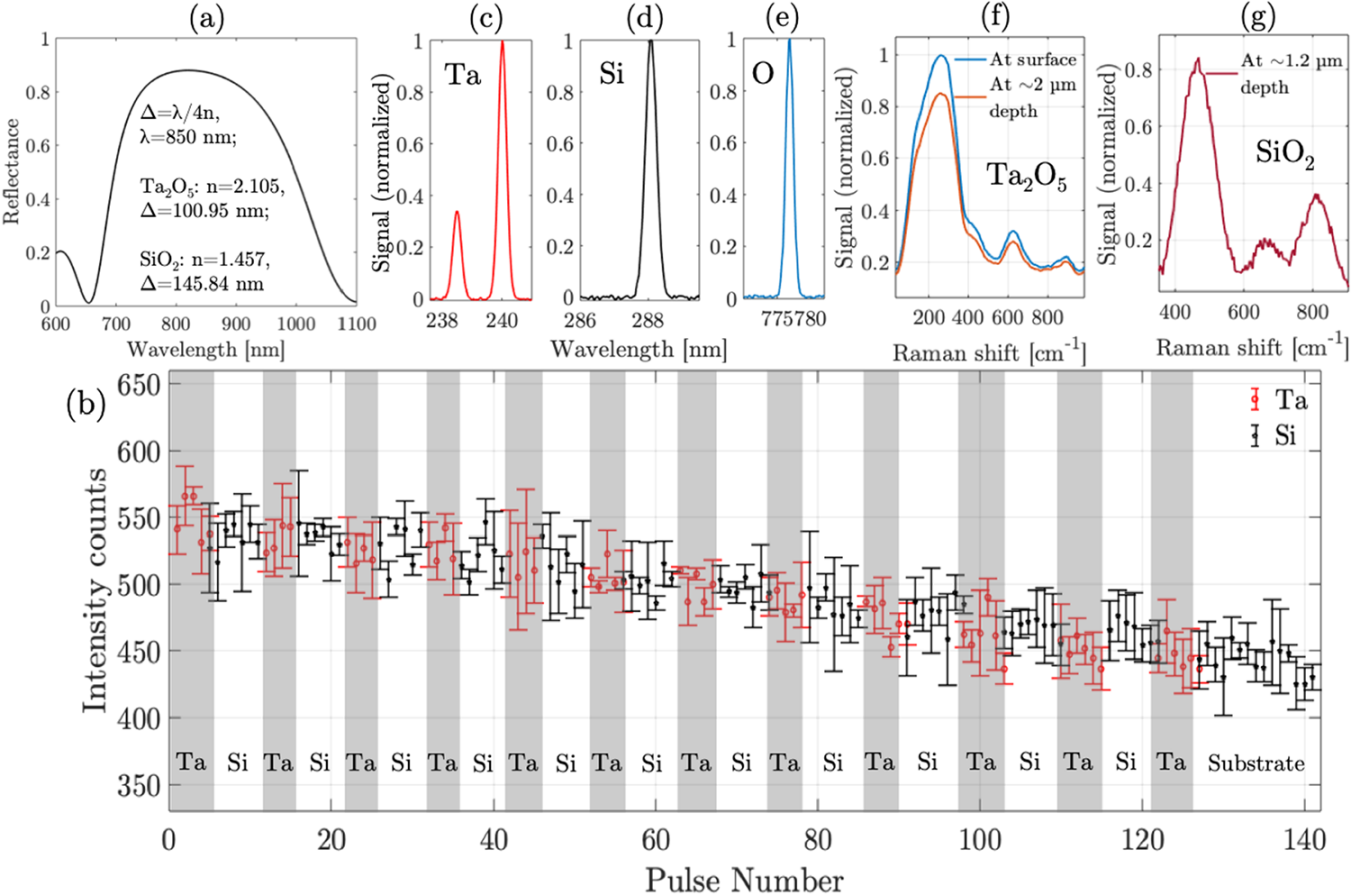
(a) Reflection simulations using KLA software,^36^ (b) LIBS signal vs pulse number, (c) LIBS for tantalum, (d) silicon, and (e) oxygen; (f, g): Raman measurements detecting spectra of (f) Ta_2_O_5_ and (g) SiO_2_.

The peak intensities of LIBS spectra are recorded for both tantalum and silicon over multiple measurements (to obtain measurement statistics and ensure repeatability), as shown in Figure S3. The LIBS of tantalum is detected at 240 nm (Figure 8c), and the LIBS of silicon is detected at 288 nm (Figure 8d). The average values of the measurements based on Figure S3, along with the errors bars representing the standard error from the mean, are plotted versus the pulse number as shown in Figure 8b. The observed standard error in the measurements in Figure 8b are ±50 counts, which can be attributed to the noise in the spectrometer and any shot-to-shot fluctuations over the multiple measurements. These errors are within the experimental error and it allows the measurements to be reproducible in terms of layer detection and profiling.

In Figure 8b, the tantalum measurement is represented by a red circle, and the silicon measurement is represented by a black star. The first few pulses 1–5 (indicated by red circles) excite the top layer of the coating, measuring the LIBS peaks of tantalum, revealing the presence of the Ta_2_O_5_ layer. The next few pulses 6–11 (indicated by black stars) detect the LIBS peaks of silicon, revealing the presence of the SiO_2_ layer. For reference, to valide the oxide layer, the oxygen peak is also detected at 777 nm, as shown in Figure 8e. Following silicon, the pulses from 12–15 again detect the LIBS peaks of tantalum, and this alternating pattern, as shown in the plot of peak measurements in Figure 8b, continues with ≈5 pulses measuring the tantalum peak, and ≈7 pulses measuring the silicon peak. After 127 pulses, the tantalum peak is no longer observed, while the silicon peak continues, potentially due to the presence of the substrate Borosilicate 33, rich in SiO_2_ (88%). Based on the simulation in Figure 8a, the tantalum and silicon layer detected with respectively 5 and 7 pulses on average should correspond to a layer thickness of 100.95 and 145.84 nm, which calculates the depth of ablation per pulse on average at ≈20–21 nm, which corresponds to the depth profiling resolution as observed above. The reduction in the peak intensities by ≈20% could be attributed to the scattering of the laser due to crater formation with successive pulses.

The presented instrument is also capable of providing Raman measurements, which can be used for depth profiling. We expect the Raman signal to be weaker than LIBS, but Raman allows the detection of the chemical composition of the layer instead of only the elemental profile. So, next, we describe the use of the same instrument to perform Raman measurements and detect the chemical composition at the ablated depths. These Raman measurements were made simultaneously with the LIBS measurements. Particularly, deep-UV Raman is useful for profiling of nanoscale thin layers, as most materials show shallow absorption depths (∼ a few 10s of nm) over deep-UV wavelengths, leading to precise Raman probing limited to the thin layer. For the experiments, the Raman measurements are achieved by attenuating the laser average power at the optical head below 2 mW. Also, an edge filter, Semrock LP266, is used at the Y2-end of the fiber to block the laser light. The spectrometer integration time is set to 10 s, with an averaging of 10 times, over a slit width of 100 μm (in order to improve the sensitivity of detection). As shown in Figure 8f, the constituent Raman peaks of Ta_2_O_5_ both at the surface and at a depth of ∼2 μm (after 100 pulses of ablation) are detected at Raman wavenumbers of 247, 631, and 851 cm^–1^, representing the Ta–O–Ta bond vibrations, validating the presence of Ta_2_O_5_ layer. When Raman measurements are made at a different depth of ∼1.2 μm (after 55 pulses of ablation), the constituent Raman spectra of SiO_2_ are detected as shown in Figure 8g, with prominent broad peaks in the range of 450–500 and 800 cm^–1^ representing Si–O–Si vibrations. As a note, to improve the sensitivity of detection of Raman spectra for SiO_2_, the spectrum in Figure 8g was obtained by summing multiple scans (∼100), along with the application of a noise filter to improve the SNR. The broadness of the spectra in both Figure 8f,g is attributed to the slit size used for sensitive detection of the Raman signal. These Raman measurements support the detection of different oxide layers in the dielectric mirror. Additionally, these measurements also demonstrate that the optical head can perform deep-UV Raman detection during the depth profiling of nanoscale multilayers, adding to the novelty of the instrument.

### c Oxide Layer Detection

A further experiment was performed to detect the native oxide layer of a silicon wafer using the deep-UV LIBS technique. The native oxide layer typically has a thickness in the ∼1–2 nm range on the surface of a silicon wafer.^37^ In this case, too, the laser was operated at a 1 Hz repetition rate. The LIBS spectrum was measured by a spectrometer (Stellarnet, Silver Nova 25, spectral resolution = 1 nm, wavelength range = 190–1100 nm, integration time = 1 s) by synchronizing with the laser pulse, with an initial delay of 1 μs. A long-wavelength range spectrometer was used to detect the LIBS peak of oxygen at 777 nm. With 1 pulse ablation, the spectrum measured around 777 nm (±10 nm) wavelength range results only in a noise floor, as shown in Figure 9a. The nanoscale thickness (∼1–2 nm) of the oxide layer on a wafer surface means that the ablation effect of 1 pulse at a single location buries the weak intensity oxygen peak in the noise floor. So, this 1 pulse ablation (followed by spectral measurement) is performed over more spatial locations (up to 100) to sum the spectra over more locations. The results are shown in Figure 9b–d, respectively, for summed spectra over 10, 50, and 100 spatial locations. It could be observed that as the spectra are summed, and with summation over >50 spatial locations, the LIBS spectral peak of oxygen at 777 nm becomes distinct in comparison to the noise floor. This demonstrates that the optical head, supported by spatial summing, can also detect nanoscale thin oxide coatings (∼1–2 nm) on top of commonly used silicon wafers. This presents a significant novelty of the deep-UV LIBS technique to be able to detect 1–2 nm thin oxide films. The method of summing the signal from spatial locations, combined with lowering the laser ablation power, can be applied for multilayered films, which would further improve the depth resolution.

**Figure 9:**
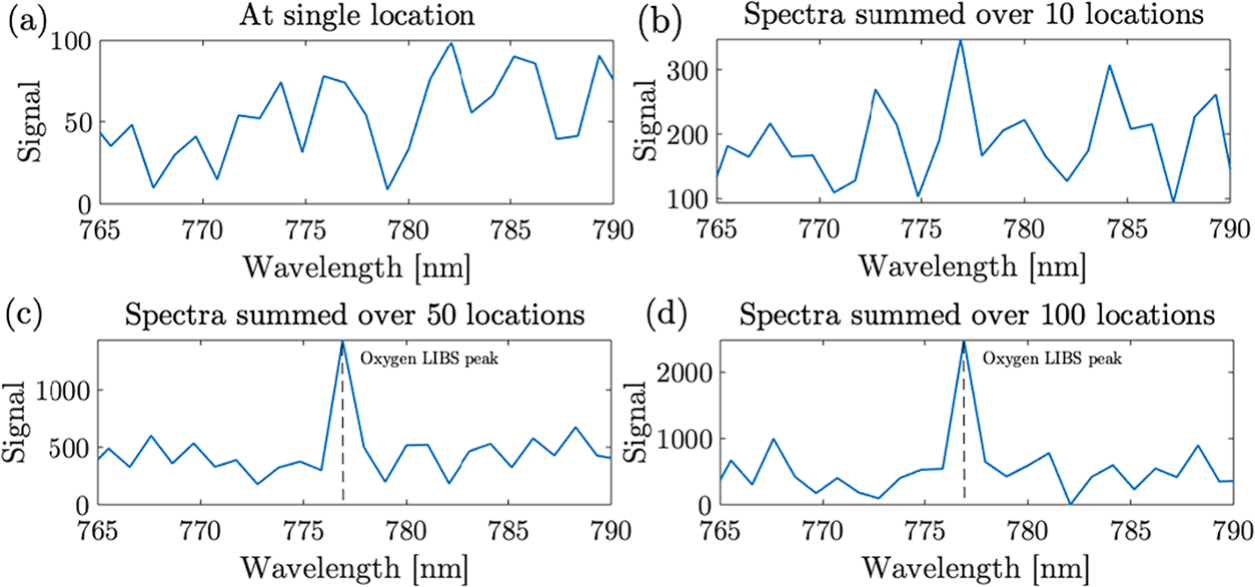
LIBS spectrum for the detection of oxygen due to the oxide layer (∼ a few nm) in a silicon wafer. The spectrum is summed over various spatial locations with values of: (a) 1, (b) 10, (c) 50, (d) 100. Resolution = 1 nm, Int. time = 1 s, Avg. = 1.

## IV Discussion

The deep-UV LIBS measurements reported here demonstrate that 266 nm excitation combined with a tightly focused beam can produce per-pulse material removal on the order of ≈20–25 nm. This enables depth resolution at a scale that is an order of magnitude (or more) finer than typical visible/NIR LIBS under comparable conditions. Practically, this depth resolution permits mapping of graded dopant profiles (e.g., the boron diffusion region in the PN junction) and resolving alternating dielectric coating layers (≈100–145 nm) with a single, ambient-condition instrument. Agreement between our LIBS-derived boron profile and a Fick’s diffusion model indicates that LIBS reliably captures the shape and characteristic diffusion length of the dopant distribution. However, converting integrated LIBS line intensities to volumetric concentrations requires independent knowledge of the mass removed per pulse and of the emission yield for the trace element in the sample. With suitable calibration, LIBS can move from relative profiling toward quantitative estimates valuable for process control and rapid screening in semiconductor and photonic device fabrication environments.

For the sensitivity with the current instrument, we noted the values as follows. The phosphorus concentration reported for the PN-junction is approximately 10^16^ cm^–3^ and the boron concentration is approximately 10^19^ cm^–3^. Using a silicon atomic density of about 5 × 10^22^ cm^–3^, these concentrations correspond to atomic fractions near 2 × 10^–7^ for phosphorus and 2 × 10^–4^ for boron. Expressed in parts per million, phosphorus at 10^16^ cm^–3^ is about 0.2 ppm and boron at 10^19^ cm^–3^ is about 200 ppm. The observation of phosphorus-level signals near the 10^16^ cm^–3^ range indicates parts-per-million sensitivity under our experimental conditions, which shows that our instrument operates in a sensitivity regime that is useful for many process-monitoring tasks.

A particularly notable result is the detection of a native silicon oxide layer approximately 1 to 2 nm thick. The oxygen emission near 777 nm became distinct from the noise floor when we spatially summed roughly 50 single-pulse spectra collected from adjacent positions. Averaging across 50 spectra improves signal-to-noise by a factor on the order of the square root of 50, which is about 7, and this improvement explains how such an ultrathin oxide can be detected under ambient conditions.

A few practical limitations of the LIBS technique became clear from the results. Although each pulse removes only ≈20–25 nm, and shows ppm sensitivity, the minimum effective lateral sampling area is limited by the numerical aperture and other optical aberrations provided by the ball lens. The measured laser-focused spot size (1/*e* ^2^) was 38 μm. By using tighter focusing or near-field/immersion optics, improvements in lateral resolution can be achieved. But this would reduce the ablated mass, and therefore the LIBS signal will also be reduced, so there is a trade-off between spatial resolution and detection sensitivity. Plasma shielding would reduce the crater size, and near-surface material ejection can further broaden the effective sampling footprint.

SIMS routinely achieves sub-10 nm depth resolution and, for many elements, it provides lower detection limits (often down to ppb), but it requires vacuum and long measurement times on a laboratory instrument. By contrast, the LIBS head works in air and gives rapid, site-selective profiles with ≈20–25 nm per-pulse depth steps and ppm-level sensitivity. SIMS is suitable only for vacuum-compatible materials.

## V Conclusions

In this work, we have demonstrated that deep-UV (266 nm) based laser-induced breakdown spectroscopy can achieve nanoscale elemental depth profiling under ambient conditions, with ablation depth per pulse as low as ≈20–25 nm and detection sensitivity of better than parts per million. By integrating a compact, fiber-coupled laser system with a miniature ball lens and autofocus system, a high depth resolution with true portability could be achieved, enabling rapid, in situ analysis without vacuum or extensive sample preparation. By applying this approach to a silicon PN junction, we obtained the boron dopant profile with depth resolution sufficient to map the ∼650 nm diffusion layer in a photovoltaic wafer, matching expected diffusion lengths and demonstrating strong agreement with Fick’s diffusion model. The ability to track the boron signal decay and the subsequent emergence of phosphorus signatures highlights the technique’s quantitative potential for semiconductor junction characterization. We further extended our measurements to a multilayer dielectric mirror, revealing alternating Ta_2_O_5_ and SiO_2_ coating layers of approximately 100 and 145 nm thicknesses, respectively. This illustrated the method’s applicability to thin-film photonic structures. Complementary Raman measurements were also demonstrated, which enabled both elemental and chemical nanoscale depth profiling with the same instrument. By spatial summing of the measured spectra with ∼50 spatial locations, the deep-UV LIBS method was also successfully applied to detect the LIBS peak of oxygen at 777 nm wavelength, from a nanoscale (∼1–2 nm) thin native oxide film on top of a silicon wafer. This shows that deep UV LIBS can be used to detect nanometer-thin films on substrates.

These results highlight the practical advantages of deep-UV LIBS. The technique delivers rapid, in situ profiling without vacuum or extensive sample preparation, which makes it well suited for high-throughput screening and process-control tasks. A concrete example is that the technique could be integrated into a semiconductor fabrication line to provide rapid, in situ feedback on the dopant diffusion depth after annealing, potentially reducing the reliance on time-consuming ex-situ SIMS analysis and improving the process control. The LIBS instrument can be combined with Raman capability in a single compact head, enabling simultaneous elemental and chemical interrogation of multilayer photonic and electronic structures, increasing diagnostic information per measurement.

At the same time, the work exposes clear limitations that must be addressed before quantitative deployment in the future. The lateral resolution of the LIBS sampling is limited by the numerical aperture and optical aberrations in the focusing lens. This limits the capability to probe submicron-scale lateral areas on the sample. Achieving true quantitative results with deep-UV LIBS will require careful calibration. It would be needed to measure the mass of the material that is removed by each laser pulse (using profilometry of single pulse craters), determine the emission yields of each element in its specific matrix, and monitor shot-to-shot fluctuations in laser energy and detector response so that measurement uncertainty can be reported. Without these steps, effects such as material redeposition, crater shape, and matrix differences can shift the apparent signal strength and bias the calculated concentrations, even though the relative depth profiles, such as the fitted Fick diffusion curve, remain reliable. Future work will therefore focus on improving spectral calibration for absolute quantification and extending the method to new materials, including perovskites and high-k dielectrics.

Moving to ultrashort pulsed lasers, such as of femto or pico-second durations, improves depth resolution because it allows nonthermal ablation giving smaller, cleaner craters and less melt redeposition. But this brings clear trade-offs: higher cost and larger, less portable systems; more sensitive alignment and optics; and higher maintenance due to UV damage or solarization. Practically, then, ultrafast deep-UV systems are best positioned as laboratory metrology tools for cross-calibration and highest-precision studies, while a calibrated nanosecond deep-UV optical head is the more pragmatic route for fast, in-line process monitoring.

## Supplementary Material



## Notes

CAD diagram of the optical head; the data for depth profiling of the PN junction and the dielectric mirror (PDF) are provided in the supporting information section.
