# Supplementary material for: Nanoscale Depth Profiling of Optoelectronic Devices Using Deep-UV LIBS
Source: ACS Omega. 2025 Nov 21;10(48):59242–50. doi: 10.1021/acsomega.5c08550 (PMC12771208; doi:10.1021/acsomega.5c08550)
Supplement: Supplementary file 1 [file ao5c08550_si_001.pdf]

Supplementary for

Nanoscale Depth Profiling of Optoelectronic Devices using Deep-UV LIBS

Atchutananda Surampudi and Mool. C. Gupta, *Fellow, IEEE.*
Charles L. Brown Department of Electrical & Computing Engineering, University of Virginia, U.S.A.

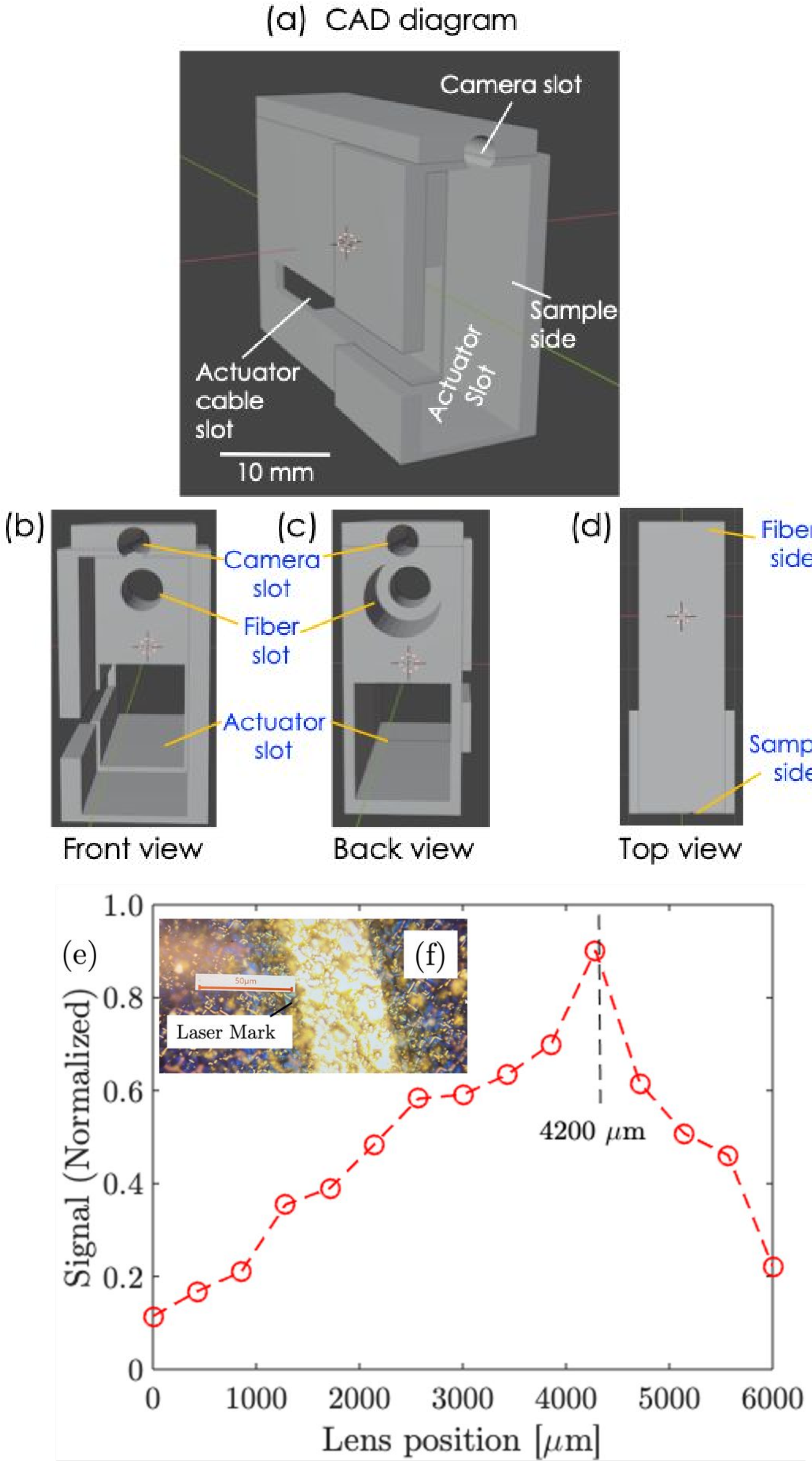

Figure S1. CAD diagram of optical head: (a) CAD diagram, (b) front view, (c) back view, (d) top view. (e) Autofocus measurements with lens position, (f) laser mark.

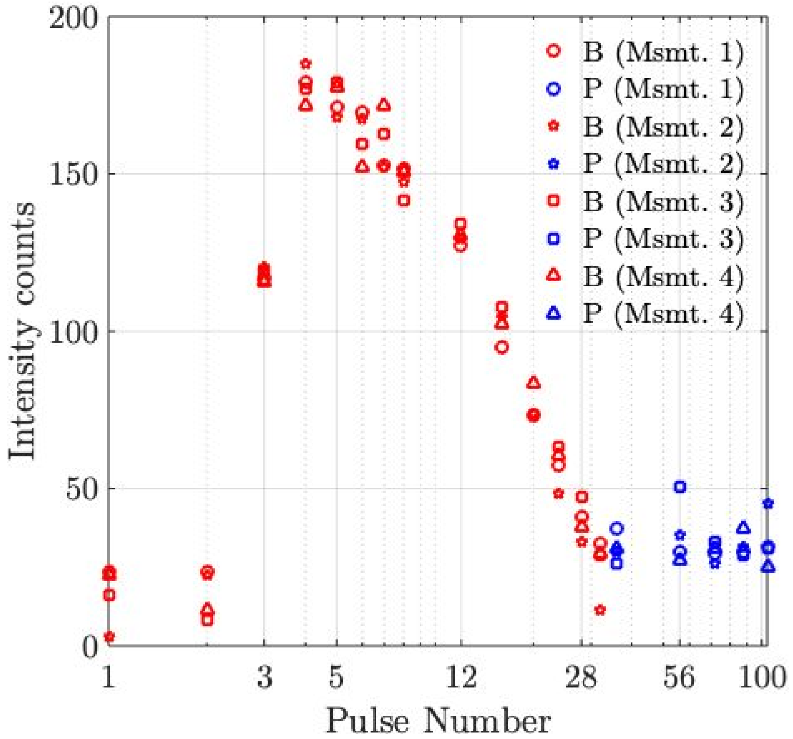

Figure S2. Data for multiple measurements (Msmt.) of PN junction depth profiling.

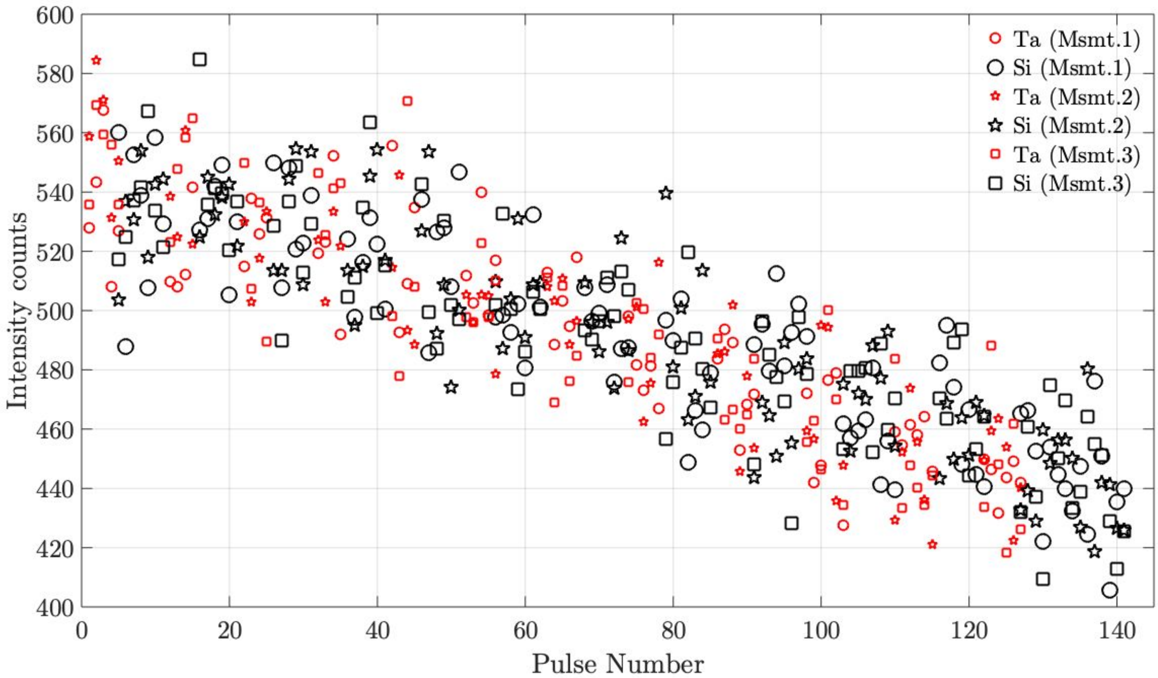

Figure S3. Data for multiple measurements (Msmt.) of dielectric mirror depth profiling.
